# A compact von Hámos spectrometer for parallel X-ray Raman scattering and X-ray emission spectroscopy at ID20 of the European Synchrotron Radiation Facility

**DOI:** 10.1107/S1600577522011171

**Published:** 2023-01-01

**Authors:** Ch. J. Sahle, F. Gerbon, C. Henriquet, R. Verbeni, B. Detlefs, A. Longo, A. Mirone, M.-C. Lagier, F. Otte, G. Spiekermann, S. Petitgirard

**Affiliations:** a ESRF – The European Synchrotron, 71 Avenue des Martyrs, CS40220, 38000 Grenoble, France; b Helmholtz-Zentrum Dresden-Rossendorf (HZDR), Institute of Resource Ecology, PO Box 510119, 01314 Dresden, Germany; c The Rossendorf Beamline at ESRF – The European Synchrotron, CS40220, 38043 Grenoble Cedex 9, France; d Department of Earth Sciences, ETH Zürich, Zürich 8092, Switzerland; ESRF – The European Synchrotron, France

**Keywords:** X-ray spectroscopy, X-ray emission spectroscopy, X-ray Raman scattering spectroscopy

## Abstract

A compact spectrometer for medium-resolution resonant and non-resonant X-ray emission spectroscopy in von Hámos geometry is described. The main motivation for the design and construction of the spectrometer is to allow for acquisition of non-resonant X-ray emission spectra while measuring non-resonant X-ray Raman scattering spectra at beamline ID20 of the European Synchrotron Radiation Facility. Technical details are provided and the performance and possible use of the spectrometer are demonstrated by presenting results of several X-ray spectroscopic methods on various compounds.

## Abstract 2

A new X-ray spectrometer in von Hamos geometry for parallel X-ray Raman scattering and X-ray emission spectroscopy is available at the inelastic X-ray scattering beamline ID20 of the ESRF.

## 1. Introduction

The arrival of the first hard X-ray free-electron lasers has led to a renewed interest in energy-dispersive spectrometers. The extremely short pulse length as well as small, yet persistent, beam jitter (in time, position, intensity and energy) still renders the use of point-by-point scanning spectrometers difficult at these new sources, therefore explaining the regained attention toward dispersive spectrometers, which enable the recording of an entire spectrum from a single X-ray pulse (Alonso-Mori *et al.*, 2012*a* ▸,*b* ▸).

Naturally, the same instruments can be used at synchrotron beamlines for hard resonant and non-resonant X-ray spectroscopy experiments, including (resonant and non-resonant) X-ray emission spectroscopy [(R)XES] and (resonant and non-resonant) inelastic X-ray scattering spectroscopy [(R)IXS]. These type of X-ray spectroscopies have found their way into physics (Herrero-Martín *et al.*, 2010 ▸; Vankó *et al.*, 2006*a* ▸), chemistry (Vankó *et al.*, 2010 ▸, 2006*b* ▸; Sá, 2014 ▸; Pollock & DeBeer, 2015 ▸), the geo-sciences (Badro *et al.*, 2003 ▸; Lin *et al.*, 2007 ▸) and biology (Lancaster *et al.*, 2011 ▸).

In the context of non-resonant IXS of hard X-rays, or X-ray Raman scattering (XRS) spectroscopy, the incident energies used to study shallow absorption edges often lie well above the *K*-shell binding energies of 3*d* transition metal elements, such as iron, cobalt and nickel, and/or above the *L*- and *M*-shell binding energies of the lanthanides and actinides. The use of a dispersive spectrometer, therefore, would allow for the measurement of non-resonant core-to-core and valence-to-core emission lines simultaneously and fully independently of the XRS signal, yielding complementary information from the low-*Z* and, for example, 3*d* transition metal elements in the investigated sample (Weis *et al.*, 2019 ▸). The most prominent geometry for dispersive X-ray spectroscopy is the von Hámos geometry (von Hámos, 1938 ▸) based on cylindrically bent analyzer crystals.

Dispersive X-ray spectrometers in von Hámos geometry exist in many shapes and sizes. Recent developments include specialized setups for multiple emission-line detection experiments (Hayashi *et al.*, 2008 ▸; Kalinko *et al.*, 2020 ▸), setups at free-electron lasers allowing high and ultrahigh time resolution (Szlachetko *et al.*, 2012 ▸; Alonso-Mori *et al.*, 2012*a* ▸,*b* ▸) as well as extreme conditions (Kaa *et al.*, 2022 ▸), and simple, yet effective, short-working-distance spectrometers (Mattern *et al.*, 2012 ▸; Pacold *et al.*, 2012 ▸). If requirements to the spectrometer energy resolution are slightly relaxed, high-quality non-resonant XES spectra can be obtained in the laboratory using cylindrically bent highly annealed pyrolitic graphite as analyzer crystals (Anklamm *et al.*, 2014 ▸; Malzer *et al.*, 2018 ▸; Zimmermann *et al.*, 2020 ▸). Furthermore, von Hámos spectrometers are important diagnostic tools at plasma sources (Shevelko *et al.*, 2002 ▸; Notley *et al.*, 2006 ▸).

Here, we present a new von Hámos spectrometer for beamline ID20 of the European Synchrotron Radiation Facility, optimized for non-resonant XES measurements during acquisition of XRS spectra. The new spectrometer is based on three cylindrically bent analyzer crystals with bending radius of *R* = 250 mm housed in a compact vacuum chamber for high signal-to-noise ratio and maximum ease of use and alignment.

The spectrometer, of course, also allows for other flavors of X-ray spectroscopy, such as RXES, including partial fluorescence yield or high-energy-resolution fluorescence-detected (HERFD) X-ray absorption spectroscopy (XAS) studies (Glatzel & Bergmann, 2005 ▸; Arandia *et al.*, 2023 ▸) and RIXS [including X-ray magnetic circular dichroism (XMCD and RIXS–MCD) experiments (Sikora *et al.*, 2012 ▸)]. However, even though feasible in von Hámos geometry, Johann or Johansson spectrometers may be better suited for resonant techniques (RXES, RIXS) and partial-fluorescence-yield measurements (HERFD XAS).

Here, we present the design and performance of the spectrometer and show several examples of applications.

## 2. Technical details

**Figure 1:**
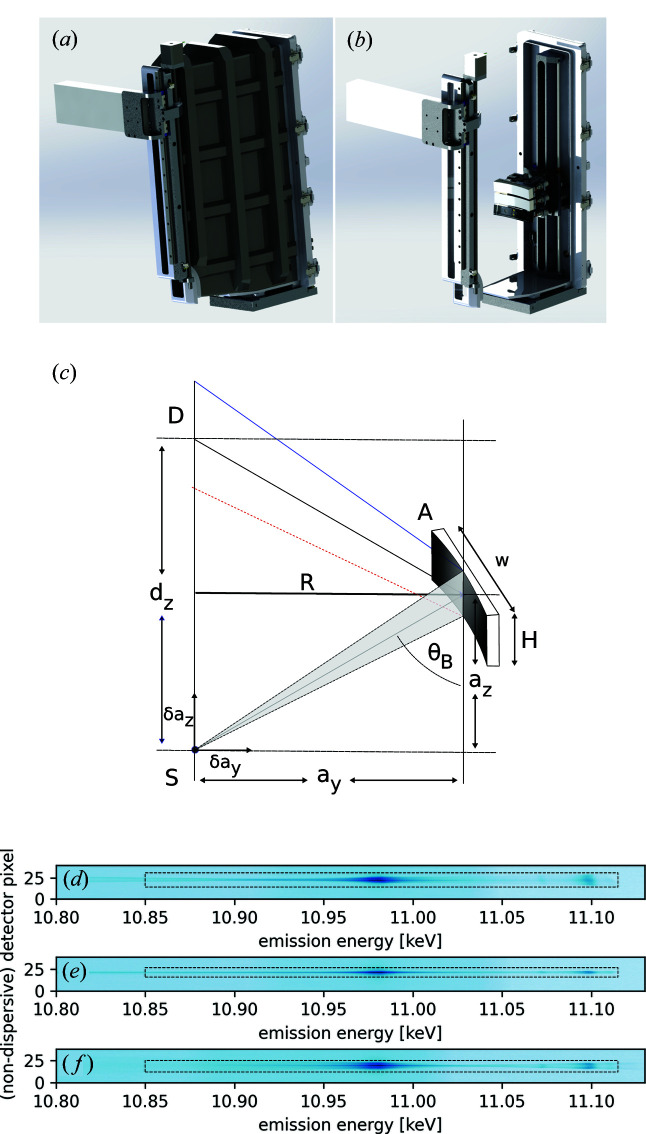
(*a*, *b*) Three-dimensional renderings of technical drawings of the von Hámos spectrometer. (*a*) An orthographic view of the entire chamber including the used Maxipix pixel detector. (*b*) Same as (*a*) but with a transparent vacuum chamber. (*c*) A schematic drawing of the von Hámos geometry; for clarity only the central analyzer crystal is shown (S: source/sample; *R*: crystal-analyzer bending radius; D: detector; *d*_*z*_: detector position; A: analyzer; *a*_*z*_: vertical analyzer position; *a*_*y*_: horizontal analyzer position; 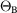 : Bragg angle; *H*: analyzer height; *w*: analyzer width; δ*a*_*z*_: vertical deviation from the ideal sample position; δ*a*_*y*_: horizontal deviation from the ideal sample position). (*d*)–(*f*) Sections of typical detector images showing the footprints of non-resonant XES from a germanium single-crystal sample. Possible regions of interest are indicated with thin dashed black lines. Images are shown on a logarithmic intensity scale.

Figs. 1 ▸(*a*) and 1 ▸(*b*) show 3D-rendered representations of technical drawings of the entire von Hámos spectrometer. We followed four principle ideas during the design of the spectrometer: compactness, portability, optimized signal-to-noise ratio, and maximum ease of spectrometer alignment and use.

The entire spectrometer consists of a single lightweight composite carbon-fiber vacuum chamber containing both the movable three-analyzer-crystal goniometer array as well as the motorized detector. The use of a vacuum chamber helps to minimize absorption and parasitic scattering from air (the typical used pressure is ∼10^−2^ mbar). The rigid chamber furthermore simplifies the alignment of the spectrometer at different spectrometer energies since both the analyzer and detector translations are motorized. This rigid design also simplifies the adjustment of the sample-to-analyzer direction such that it lies parallel to the polarization vector of the incident X-ray beam. Due to the finite size of the vacuum chamber, analyzer Bragg angles in the the range of *ca.* 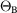 = 45–85° are available.

As mentioned, the goniometer array allows for three cylindrically bent crystal analyzers with a meridional bending radius of *R* = 250 mm. Each analyzer crystal is 25 mm high and 110 mm wide. At present, Si(*n*,*n*,*n*), Si(*n*,*n*,0) and Si(*n*,0,0) analyzer crystals are available. The three analyzer crystals are mounted on a common translation axis parallel to the central analyzer’s cylinder axis. The cylinder axes of the outer two crystals are tilted by ±5° with respect to that of the central analyzer to roughly allow the same range of Bragg angles as the central analyzer crystal for a common detector position (Alonso-Mori *et al.*, 2012*a* ▸). This, however, results in the fact that only the central analyzer crystal produces a perfect focus across the entire dispersion footprint on the detector surface [compare Figs. 1 ▸(*d*)–1 ▸(*f*)]. The analyzer goniometer array allows for three individual degrees of motion for each analyzer crystal: 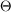 and χ rotation, as well as translation along the focus direction.

The X-rays are detected by a 5 × 1 Maxipix hybrid pixel detector (500 µm sensor thickness) with 55 µm × 55 µm pixel size and 14 mm × 70 mm active surface area (Ponchut *et al.*, 2011 ▸). Sections of typical detector images showing the X-ray emission spectrum of a germanium single-crystal sample as a footprint for each of three Si(6,6,0) analyzer crystals are shown in Figs. 1 ▸(*d*)–1 ▸(*f*). Possible regions of interest are shown as thin dashed black lines.

The solid angle covered by the spectrometer is given by

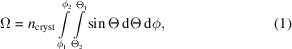

where *n*_cryst_ is the number of analyzer crystals and the integral limits are defined by the extent of each of the analyzer crystals and their orientation inside the spectrometer chamber. For the presented spectrometer, the solid angle is of the order of 1% of 4π (energy integrated).

**Figure 2:**
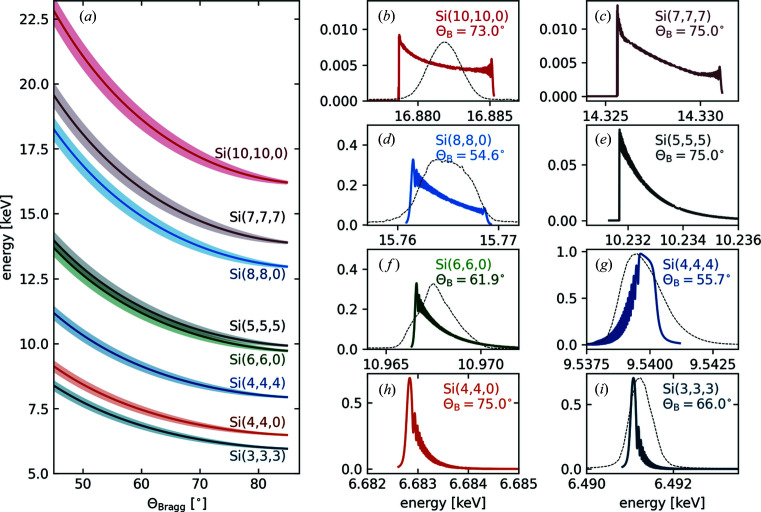
(*a*) DuMond diagram for bent *R* = 250 mm Si crystals for different (*n*,*n*,0) and (*n*,*n*,*n*) lattice planes. Shaded areas represent the energy window covered by the analyzer crystals. (*b*)–(*i*) Calculated (colored) and experimental (thin dashed) reflectivity curves between *ca*. 6 and 17 keV. All theoretical reflectivity curves were obtained by solving the 1D Tagaki–Taupin equations (Takagi, 1962 ▸, 1969 ▸; Taupin, 1964 ▸) using the *pyTTE* package by Honkanen & Huotari (2021 ▸).

The energy dispersion and energy window simply follow from Bragg’s law,

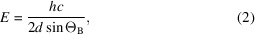

and the spectrometer geometry,

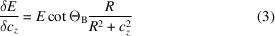

where *R* is the cylindrical curvature of the analyzer crystals, *E* is the central energy and *c*_*z*_ is the vertical analyzer coordinate. The dispersion relation as well as the energy windows covered (shaded areas) for several reflections of the Si(*n*,*n*,*n*) and Si(*n*,*n*,0) lattice plane family over the available angular range of the spectrometer are shown in Fig. 2 ▸(*a*).

## 3. Energy resolution

The overall energy resolution of the setup is determined by the bandwidth of the incident X-ray photon beam Δ*E*_in_, the bandwidth of the analyzer crystals Δ*E*_TT_, and the source size contributions in the horizontal Δ*E*_*y*_ and vertical Δ*E*_*z*_ directions,

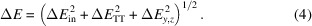

Ideally, the overall beamline and spectrometer resolution should be comparable with the lifetime broadening induced by the created core hole. In the case of non-resonant XES, the requirements are further relaxed as the incident X-ray bandwidth does not contribute.

The contribution of the analyzer crystals is given by the width of their X-ray reflectivity curve for the used Bragg reflection. For cylindrically bent crystals these curves can be approximated by solving the Tagaki–Taupin equations (Takagi, 1962 ▸, 1969 ▸; Taupin, 1964 ▸). A comparison of these theoretical Tagaki–Taupin reflectivities and experimentally measured elastic lines for different analyzer crystals and reflections are shown in Figs. 2 ▸(*b*)–2 ▸(*i*). All experimental curves were measured using an Si(3,1,1) channel-cut post-monochromator providing a resolving power of Δ*E*/*E* ≃ 2.8 × 10^−5^ for the incident X-ray beam. The Tagaki–Taupin equations were solved using the *pyTTE* package by Honkanen & Huotari (2021 ▸). Compression stress, as discussed by Honkanen *et al.* (2014 ▸, 2017 ▸) for cylindrically bent crystals, or finite analyzer size are not accounted for in the presented simulated reflectivity curves.

The source size contribution follows from the dispersion relation [equation (3)] and the variation of the Bragg angle 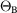 as a function of the horizontal and vertical deviation from a perfect point source, 

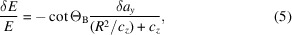

and 

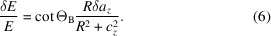




The finite detector pixel size *p* contributes via a dispersion similar to equation (3) if *c*_*z*_ is replaced by *d*_*z*_ = 2*c*_*z*_, the detector coordinate. This contribution is of the order of a few hundred meV at large 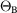 . Here, we have not explicitly considered this contribution as the pixelated area detector can be translated parallel to the analyzer cylinder axis, and several detector exposures at different vertical positions incommensurate with the detector pixel spacing can be combined to a virtual arbitrary pixel size along the dispersive direction. The translation along the dispersive direction furthermore enables the recording of smooth and continuous spectra even at emission energies that correspond to the detector-chip corners. The achievable overall energy resolution is well below the broadening resulting from the finite lifetime of deep core holes in intermediate-*Z* elements.

## 4. Examples

### 4.1. Non-resonant X-ray emission spectroscopy

**Figure 3:**
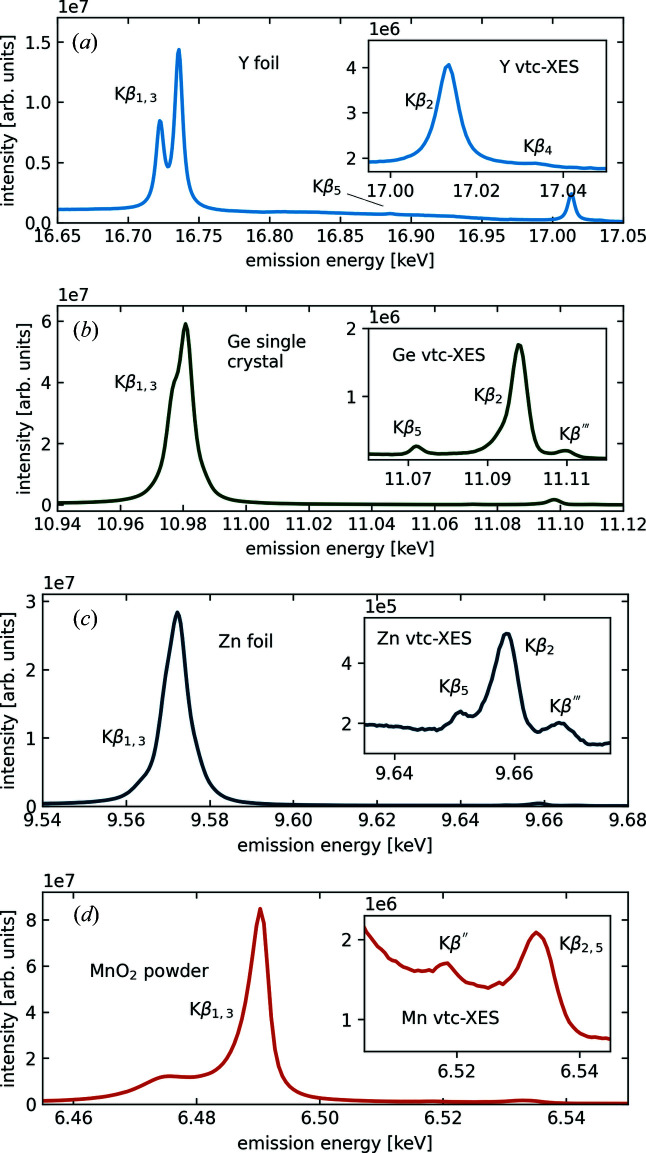
Examples of non-resonant *K*β XES spectra. The insets show the valence-to-core (vtc) regions of the respective spectra. (*a*) An yttrium *K*β XES spectrum of an yttrium foil. (*b*) A Ge *K*β XES spectrum of a Ge foil. (*c*) A Zn *K*β XES spectrum of a polycrystalline ZnO powder sample. (*d*) A Mn *K*β XES spectrum of a polycrystalline MnO powder sample.

Fig. 3 ▸ shows examples of non-resonant *K*β core-to-core and valence-to-core X-ray emission lines of different elements. The spectra were taken with incident energies 200 eV above the respective absorption edge and exposure times of 7 min per spectrum. The photon flux at the sample was 1 × 10^13^ photons s^−1^ within a 10 µm × 10 µm spot size. Fig. 3 ▸(*a*) shows the Y *K*β emission line from a 25 µm-thick yttrium foil measured using the Si(8,8,0) reflection at a mean Bragg angle of 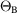 = 50° [see Fig. 2 ▸(*d*)]. The signal from all three analyzer crystals was averaged over. Fig. 3 ▸(*b*) depicts a spectrum of the *K*β and valence-to-core emission line of Ge from a millimetre-sized Ge single-crystal sample using three Si(6,6,0) analyzer crystals with a central Bragg angle of 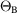 = 61° [resolution function shown in Fig. 2 ▸(*f*)]. Fig. 3 ▸(*c*) shows *K*β and valence-to-core emission lines of Zn from a 25 µm-thick Zn foil. Here, the spectra represent averaged signals from three Si(4,4,4) analyzer crystals at a mean Bragg angle of 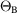 = 55° [resolution function shown in Fig. 2 ▸(*g*)]. The *K*β and valence-to-core emission lines of Mn from a small pellet of polycrystalline MnO_2_ (grain size <10 µm) are shown in Fig. 3 ▸(*d*) [resolution function shown in Fig. 2 ▸(*i*)]. We used a single Si(3,3,3) analyzer crystal at a central Bragg angle of 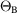 = 65°.

These examples show that high-quality non-resonant core-to-core and valence-to-core XES spectra over the entire energy range available at ID20 can be measured on a minute timescale, which renders this setup perfectly suitable for simultaneous measurements of XRS spectra of low- and intermediate-*Z* elements and non-resonant XES from, for example, 3*d* transition metals.

### 4.2. Combined XRS and XES

One of the main motivations driving the development of this compact and mobile von Hámos spectrometer is the prospect of studying non-resonant X-ray emission lines while measuring non-resonant IXS spectra of shallow bound electrons, so-called XRS spectroscopy. The hard X-rays used for XRS spectroscopy are often well above, for example, the *K*-shell binding energies of 3*d* transition metals or the *L*-shell binding energies of 5*d* and 4*f* elements. Among the first of such combined XES and XRS experiments, Weis *et al.* (2019 ▸) simultaneously measured the Fe *K*β_1,3_ and Fe valence-to-core-emission lines via non-resonant XES and the Fe *M*_2,3_-edge via XRS in order to study the pressure-induced spin transition in siderite (FeCO_3_).

**Figure 4:**
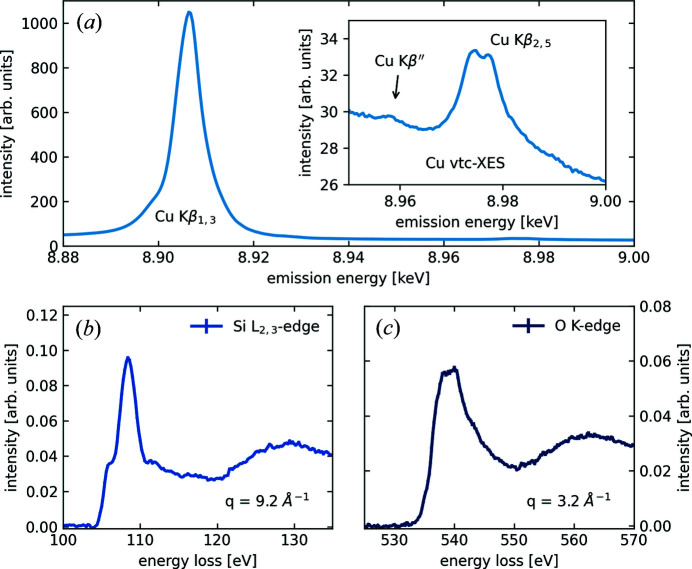
(*a*) A non-resonant X-ray emission spectrum of the Cu *K*β emission line, as well as the valence-to-core part of the same emission line of a copper-exchange zeolite. (*b*) An Si *L*_2,3_-edge excitation spectrum measured at high momentum transfer *q* and (*c*) an O *K*-edge spectrum at low momentum transfer of the same sample. The XES spectra were measured simultaneously to the two XRS spectra.

Fig. 4 ▸ shows simultaneously measured XES and XRS spectra of a copper-exchanged zeolite with chabazite topology at ambient conditions. This zeolite and similar copper-exchanged molecular sieves are promising catalyst candidates for the direct conversion of methane to methanol (Borfecchia *et al.*, 2018 ▸). Despite recent advances, key research questions remain open about the chemical identity and electronic structure of Cu and/or participating oxygen species formed during thermal treatment in an oxidizing atmosphere. These investigations are challenging based on Cu *K*-edge XAS alone (Borfecchia *et al.*, 2015 ▸; Martini *et al.*, 2017 ▸; Pappas *et al.*, 2017 ▸, 2018 ▸). Simultaneously measured XES and XRS offer the necessary bulk sensitivity to probe these intermediates dispersed in the whole microporous solid and, in addition, are a unique opportunity to access element-selective information on extra-framework O and C species evolving during the reaction. Fig. 4 ▸(*a*) shows the Cu *K*β emission line and the zoomed-in valence-to-core region in the inset. The Cu *K*β′′ line is clearly visible at ∼8.96 keV emission energy. Figs. 4 ▸(*b*) and 4 ▸(*c*) show the Si *L*_2,3_- and O *K*-edge of the zeolite, respectively. The measurement time for the data shown in Fig. 4 ▸ was 80 min and the overall resolution for the XES setup using a single cylindrically bent Si(5,5,5) analyzer crystal was 1.5 eV. For the XRS setup with 24 spherically bent Si(6,6,0) analyzer crystals, a resolution of 0.7 eV was achieved (Huotari *et al.*, 2017 ▸).

This example shows the great potential resulting from a combination of XRS with XES to study, at the same time, low-*Z* absorption edges via XRS and heavier elements, such as 3*d* transition metal elements, via non-resonant XES. Especially in the valence-to-core region of the emission spectra, XES and XRS provide complementary information on the electronic structure of the studied sample. The advantage of this approach is that both probes make use of hard X-ray photons, which are compatible with complex sample environments such as *in situ* and *operando* cells for the study of chemical processes and reactions and/or high-pressure diamond anvil cells for the study of samples under extreme pressure conditions (Petitgirard *et al.*, 2017 ▸, 2019 ▸).

### 4.3. Resonant X-ray emission spectroscopy

The ability to record an emission spectrum at electronvolt resolution over tens of electronvolts renders the von Hámos spectrometer well suited to record entire resonant X-ray emission maps by simply varying the incident photon energy across one of the system’s resonances.

This type of spectroscopy is often also referred to as RIXS; however, here we do not show momentum-transfer resolved spectra and therefore refrain from using the term ‘scattering’.

**Figure 5:**
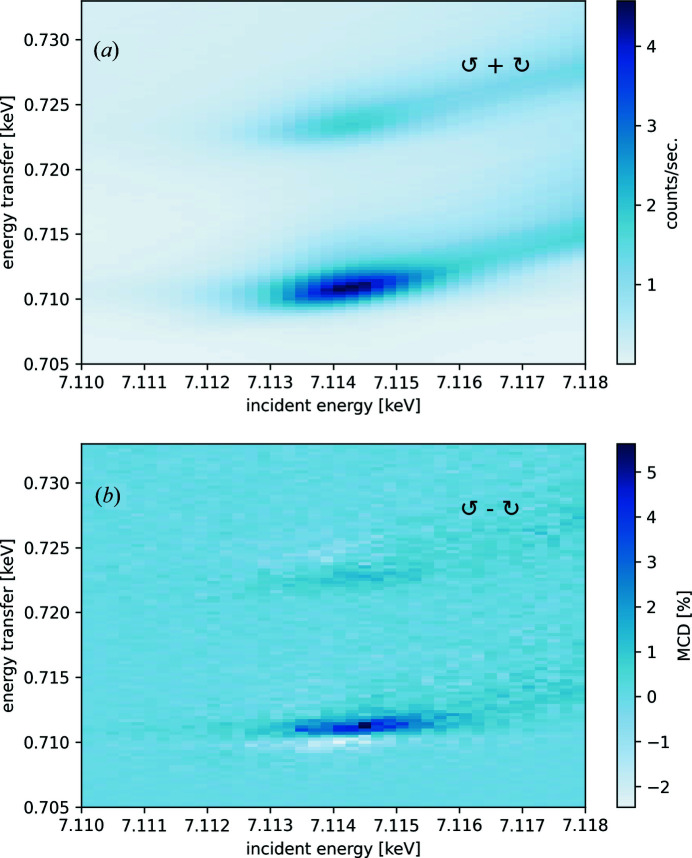
(*a*) An RXES map of an NdFeB permanent magnet measured at the Fe *K*-edge (resulting from the sum of two RXES maps measured with circular-left and circular-right polarized photons). (*b*) RXES–MCD shown as a difference map (in %) of the two RXES maps.

As an example of such resonance maps, Fig. 5 ▸ shows data at the Fe *K*-edge resonance recorded with left- and right-circularly polarized incident X-rays, as first observed at resonance by Krisch *et al.* (1996 ▸) for gadolinium. Sikora *et al.* (2010 ▸) showed the increased XMCD in the vicinity of the Fe *K*-edge for magnetite using these types of resonance maps.

Fig. 5 ▸(*a*) shows the 1*s*2*p* RXES plane measured at the Fe *K*-edge of an NdFeB permanent magnet as a result of the sum of two resonance maps recorded with circularly left- and right-polarized X-rays. Fig. 5 ▸(*b*) shows the MCD map of the same data resulting from the difference between the two RXES maps.

Here, we used X-rays from three consecutive U32 undulators and an Si(1,1,1) high-heatload double-crystal monochromator. Circular left- and right-handed polarized X-ray beams were produced using a double phase-plate setup in quarter wave-plate mode [2 × 0.6 mm-thick diamond-〈110〉 phase plates using the (1,1,1) reflection in Laue geometry]. The setup provides high-purity polarization, but at the expense of only ∼18% transmission at the Fe *K*-edge (7.112 keV). The double phase-plate configuration compensates the vertical beam divergence and energy-dispersion contribution to the depolarization of the X-ray beam (Scagnoli *et al.*, 2009 ▸). The working point orientation of the retarder plates was calibrated prior to the experiment to follow the incident photon energy for each scan. To record the emission spectra for different incident energies, we used a single cylindrically bent Si(4,4,0) analyzer crystal at a mean Bragg angle of approximately 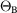 = 65°. In combination with the incident energy bandwidth from the monochromator, the overall energy resolution was 1.9 eV.

## 5. Summary and conclusions

A new compact and portable dispersive X-ray spectrometer in von Hámos geometry is available at ID20. The spectrometer is optimized for measurements of non-resonant X-ray emission spectra while acquiring X-ray Raman scattering spectra; however, it is also available for standalone resonant and non-resonant X-ray emission spectroscopy measurement. We have described the technical details of this low-noise spectrometer and have presented various examples of the use of this new spectrometer at the European Synchrotron Radiation Facility.
